# Dissociation of SHP-1 from Spinophilin during Platelet Activation Exposes an Inhibitory Binding Site for Protein Phosphatase-1 (PP1)

**DOI:** 10.1371/journal.pone.0119496

**Published:** 2015-03-18

**Authors:** Peisong Ma, Darci C. Foote, Andrew J. Sinnamon, Lawrence F. Brass

**Affiliations:** Department of Medicine and Pharmacology, University of Pennsylvania, Philadelphia, Pennsylvania, United States of America; University of Leuven, BELGIUM

## Abstract

We have recently shown that a critical regulatory node in the platelet signaling network lies immediately downstream of platelet receptors for thrombin and TxA_2_. This node is comprised of a scaffold protein (spinophilin, SPL), a protein tyrosine phosphatase (SHP-1), and either of the two members of the Regulators of G protein Signaling family predominantly expressed in platelets (RGS10 or RGS18). The SPL/RGS/SHP-1 complex is present in resting platelets, dissociating when thrombin or TxA_2_, but not ADP or collagen, activate SHP-1 and release RGS10 and RGS18 to dampen signaling. Here we demonstrate an additional regulatory role for spinophilin, showing that dissociation of SHP-1 from spinophilin is followed by an increase in the binding of spinophilin to PP1, a serine/threonine phosphatase whose binding site maps to a region close to the SHP-1 binding site. The increase in PP1 binding to spinophilin is limited to platelet agonists that cause dissociation of the complex and is selective for the α and γ isoforms of PP1. Studies in cell culture show that SHP-1 and PP1 can compete for binding to spinophilin and that binding inhibits PP1 activity since over-expression of wild type spinophilin, but not spinophilin with a disabled PP1 binding site, causes an increase in the phosphorylation of myosin light chain, a well-characterized PP1 substrate. Collectively, these results indicate that in addition to regulating RGS protein availability in resting platelets, spinophilin can serve as a time-dependent, agonist- and isoform-selective regulator of PP1, inhibiting its activity when decay of the SPL/RGS/SHP-1 complex releases SHP-1 from spinophilin, exposing a binding site for PP1.

## Introduction

With the exception of collagen, most platelet agonists work through G protein coupled receptors, invoking signaling events that lead to platelet aggregation and thrombus formation [1]. As in other cells, G protein-dependent signaling in platelets is negatively regulated by members of the RGS (regulator of G protein signaling) family, two of which, RGS10 and RGS18, are strongly expressed in human and mouse platelets [2–5]. RGS proteins help to terminate signaling by accelerating the hydrolysis of GTP by G protein α subunits [6].

In previous studies, we showed that removing the restraining influence of RGS proteins in platelets produces a gain of function *in vitro* and *in vivo* [7, 8], an effect that has now also been observed by other investigators studying mice that lack RGS18 [9]. We also showed that in resting platelets RGS10 and RGS18 are bound to a scaffold protein, spinophilin (SPL or neurabin-II), forming a complex in which spinophilin is phosphorylated on tyrosines 398 and 483 [2]. Phosphorylated Y398 provides a binding site for one of the two SH2 domains in the tyrosine phosphatase, SHP-1 [2]. Activation of SHP-1 leads to dephosphorylation of spinophilin and dissociation of the SPL/RGS/SHP-1 complex, releasing RGS10 and RGS18, which can then dampen signaling that otherwise favors platelet activation [10, 11]. Of relevance for the present studies, dissociation of the SPL/RGS/SHP-1 complex is agonist-selective, occurring in response to thrombin and thromboxane A2 (TxA2) mimetics, but not in response to ADP or collagen, neither of which signals potently via Gq [1].

Although spinophilin can bind to members of the RGS protein family [12–14], it was originally identified in rat brain as a protein that binds to the serine/threonine phosphatase, PP1 [15]. Studies using pharmacologic inhibitors [16–20] and genetic manipulation [21] suggest that PP1 promotes platelet activation and have identified phosphorylated myosin light chain (MLC) as one of its substrates [22–24]. Platelets express three isoforms of the PP1 catalytic unit, denoted α, β and γ [3–5]. PP1 substrate specificity depends on a diverse set of regulatory proteins of which spinophilin is one [25, 26]. The crystal structure of the SPL/PP1 complex indicates that binding to spinophilin should inhibit PP1 activity on some of its substrates, but not all, in part because of the existence of multiple substrate binding pockets [27].

With this background in mind, we have examined the mechanism, timing and consequences of the interaction of PP1 with spinophilin in platelets. Although SPL/PP1 interactions have been studied in other cells [15, 28–30], platelets present a challenge because the PP1 binding site on spinophilin residues 417–494 [31] is adjacent to the SHP-1 binding site identified in our earlier studies, raising the question of whether both phosphatases can bind to spinophilin at the same time and, if not, how the choice between the phosphatases is governed [2]. This is not an issue in most cell types: while PP1 isoforms are expressed ubiquitously, expression of SHP-1 is limited to hematopoietic cells and the widely-expressed non-receptor tyrosine phosphatase, SHP-2, does not bind to spinophilin [2]. Thus, in platelets and other cells that express spinophilin, PP1 and SHP-1, there is a potential competition between PP1 and SHP-1 whose timing and consequences we have considered.

Here we show that although the binding of PP1 to spinophilin can be detected at low levels in resting platelets, it greatly increases when platelets are activated by agonists that cause dissociation of the SPL/RGS/SHP-1 complex (thrombin and TxA_2_), but not those which do not (ADP and collagen). Binding to spinophilin is selective for PP1α and PP1γ, and does not occur with PP1β. Binding to spinophilin inhibits PP1 activity towards a small molecule substrate and leads to an increase in the phosphorylation of myosin light chain. Collectively, these results show that agonist-selective decay of the SPL/RGS/SHP-1 complex and formation of the SPL/PP1 complex can help to regulate platelet activation by at least two mechanisms. The first limits the duration of G protein signaling by regulating the availability of RGS10 and RGS18; the second regulates PP1 activity.

## Methods and Materials

### Materials

Apyrase and ADP were purchased from Sigma-Aldrich (St. Louis, MO), thrombin was from Haematologic Technologies, Inc. (Essex Junction, VT, USA), U46619 from CalBiochem (San Diego, CA) and collagen from Chrono-log (Havertown, PA). Goat anti-spinophilin (A-20), mouse anti-pan-PP1 (E-9), rabbit anti-PP1 (FL-18), goat anti-PP1α (C-19), goat anti-PP1β (C-20) and goat anti-PP1γ (C-19) and donkey anti-goat IgG-HRP were purchased from Santa Cruz Biotechnology (Santa Cruz, CA). Anti-pTyr Ab was from Upstate. Rabbit anti-Flag, rabbit anti-phospho-myosin light chain 2 (Thr18/Ser19), rabbit anti-myosin light chain 2, rabbit anti-β-actin, and mouse (9B11) and rabbit (71D10) anti-Myc were from Cell Signaling Technology (Danvers, MA). Mouse anti-Flag (M2) was from Sigma-Aldrich. pEx39Not+ containing the cDNA encoding rat spinophilin was a gift from Dr. Patrick Allen (Yale University). Full-length SPL was subcloned into the pCR-Blunt II-TOPO vector using the Zero Blunt TOPO PCR cloning kit (Invitrogen, Carlsbad, CA). SPL was additionally subcloned into pCMV-3B expression vector, using restriction sites for *HindIII* and *SalI* incorporated by synthetic oligonucleotides. Human SHP-1 in pCDNA3.1 was constructed from a pGEX-2T plasmid encoding a GST-SHP-1 fusion protein, which was a gift from Dr. Benjamin Neel (Ontario Cancer Institute). Human pCDNA3 Flag-PP1 α, β and γ plasmids were kindly provided by Dr. Jeong-Heon Lee (Wells Center for Pediatric Research). Spinophilin and PP1α variants were generated by site-directed mutagenesis (Agilent Technologies, Santa Clara, CA).

### Isolation of human platelets

Blood was obtained from healthy donors using protocols approved by the University of Pennsylvania IRB. Written informed consent of all donors was obtained prior to blood collection. Blood was anticoagulated 1:5 with ACD (65 mM Na_3_ citrate, 70 mM citric acid, 100 mM dextrose, pH 4.4) and centrifuged at 129xg for 20 min to obtain platelet rich plasma (PRP). Washed platelets were prepared by sedimentation at 341xg for 10 min. Platelets were washed with HEN buffer (150 mM NaCl, 1 mM Na_2_EDTA, 10 mM HEPES, pH 6.5) containing 1 μM PGI_2_ and resuspended in modified Tyrode's buffer (137 mM NaCl, 20 mM HEPES, 5.6 mM glucose, 1 g/liter BSA, 1 mM MgCl_2_, 2.7 mM KCl, 3.3 mM, NaH_2_PO_4_, pH 7.4).

### Cell culture and transfection

Chinese hamster ovary (CHO) cells (ATCC Cat#CCL-61) were maintained in Ham’s F-12 Nutrient Mixture medium (Invitrogen) supplemented with 10% fetal bovine serum, 100 U/ml penicillin and 0.1 mg/ml streptomycin. Cells were transfected at 60% confluence using X-tremeGENE reagent (Roche Applied Science). Cells were harvested and assayed 48 h after transfection. When indicated, they were serum-starved overnight starting approximately 30 hours post transfection.

### Immunoblotting

Platelets or cells were lysed in NP-40 (50 mM Tris, 150 mM NaCl, 2mM EDTA, 1mM EGTA, 1% NP40, pH 7.4) or Triton X-100 lysis buffer (50 mM Tris-HCl, 100 mM NaCl, 5 mM EDTA, 1% Triton X-100, pH 7.4) in the presence of protease inhibitors. The lysates were boiled in sample buffer before sulfate-polyacrylamide gel electrophoresis (SDS PAGE) analysis. Binding of the primary antibodies was detected using HRP-conjugated secondary antibodies and the ECL-system (Amersham Biosciences). Individual bands were quantified by densitometry and analyzed using NIH ImageJ software.

### Co-precipitation experiments in CHO cells and platelets

CHO cells were lysed 48 h after transfection in ice-cold lysis buffer (50 mM Tris-HCl, 100 mM NaCl, 5 mM EDTA, 1% Triton X-100, pH 7.4) containing complete protease inhibitor cocktail and/or 2 mM Na_3_VO_4_. Washed platelets were incubated in an aggregometer cuvette at 37°C, and lysed with ice-cold 5x Nonidet P-40 buffer (1% NP-40 in 50 mM Tris, 150 mM NaCl with protease inhibitor and 2 mM Na_3_VO_4_). When indicated, platelets were preincubated with aspirin (ASA, 1 mM for 30 min). After centrifugation at 16,000xg for 20 min at 4°C, supernatants were precleared with protein A agarose (for rabbit antibodies) or protein G agarose (for goat and mouse antibodies) for an hour and incubated overnight at 4°C with an immunoprecipitating antibody (2 μg) or normal rabbit, goat or mouse IgG. Protein/antibody complexes were isolated with protein A- or G-agarose for 2 h at 4°C. After 5 washes with lysis buffer, the beads were boiled in sample buffer (2% SDS/1% 2-mercaptoethanol/0.008% bromophenol blue/80 mM Tris/1 mM EDTA, pH 6.8).

### PP1 phosphatase activity

PP1 activity was measured with the fluorescent-based Rediplate 96 Enzchek kit R-33700 (Invitrogen, Carlsbad, CA) following the manufacturer’s protocol. Myc-SPL and/or PP1 transfected CHO cells lysates were incubated overnight at 4°C with 2 μg mouse anti-Myc antibody or normal mouse IgG. Protein/antibody complexes were isolated with protein G magnetic beads (Millipore, Temecula, CA) for 2 h at 4°C. After 5 washes with lysis buffer, the beads were added into microplate well containing 80 μl reaction buffer. The reactions were incubated at room temperature for 20 min. Fluorescence was measured at 355 nm excitation and 460 nm emission.

### Statistical analysis

Comparisons were made using Student’s t-test with P≤0.05 considered to be statistically significant.

## Results

### Spinophilin binds PP1 in platelets in an agonist-regulated and isoform-selective manner

**Fig 1:**
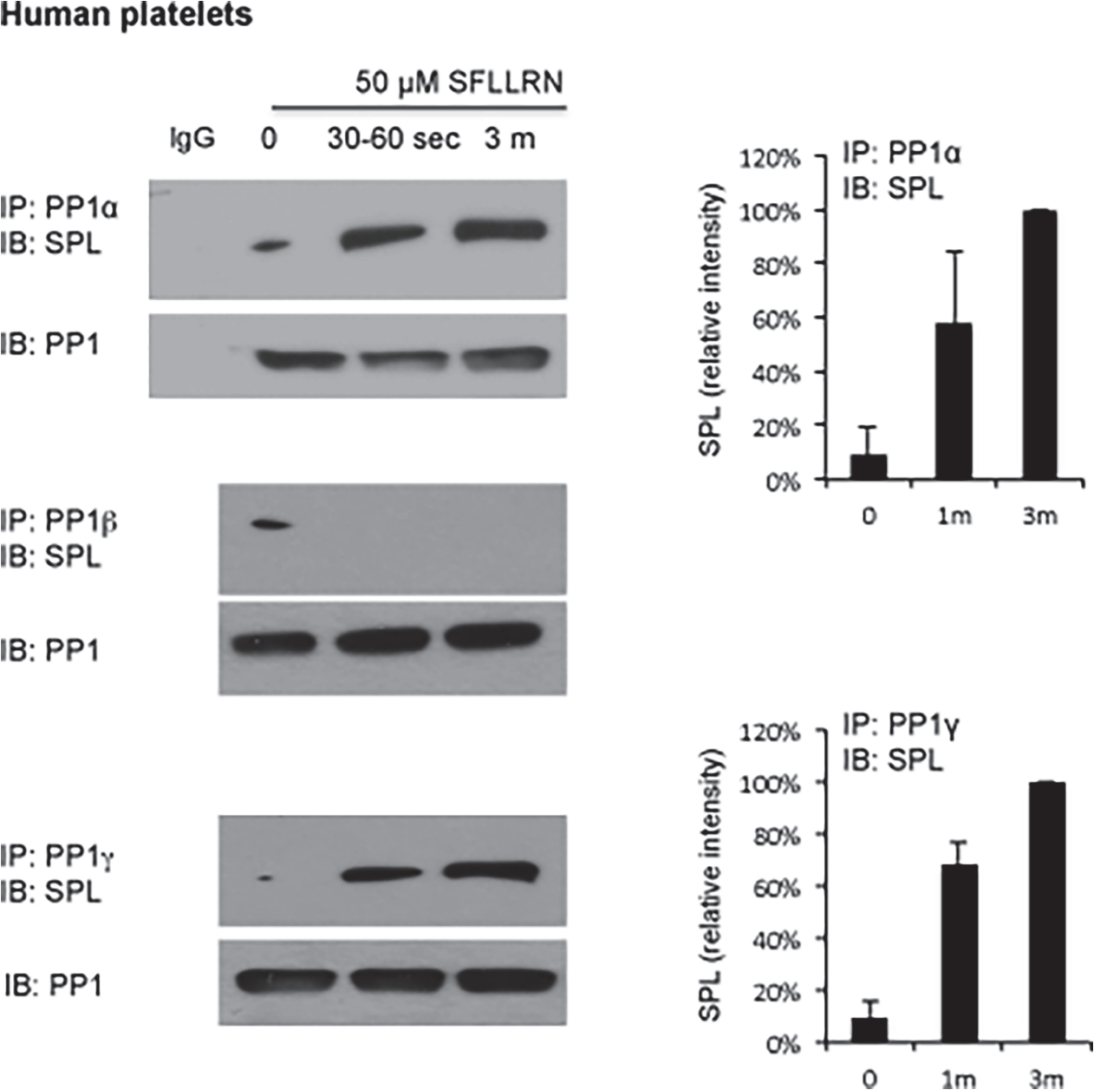
Formation of an SPL/PP1 complex in platelets. Lysates were prepared from platelets stimulated with the PAR1 agonist peptide, SFLLRN, after which PP1 precipitated with isoform-selective antibodies or nonimmune immunoglobulin (Ig) and probed with anti-spinophilin before re-probing with a pan-PP1 (E-9) antibody. The graphs for PP1α and γ summarize results from two experiments expressed as % of the result obtained at 3 min. No binding of PP1β was detectable at either time point after the addition of SFLLRN.

PP1 and spinophilin are widely expressed in human and rodent tissues, including platelets [2, 30]. Platelets express the α, β and γ isoforms of PP1 [3–5]. Co-precipitation studies using lysates from human platelets showed that there was little, if any, detectable binding of any of these isoforms to spinophilin in resting platelets. Adding the PAR1 (thrombin receptor) agonist peptide, SFLLRN, caused an approximately 10-fold increase in binding by 3 minutes after addition of the peptide that was isoform-specific, occurring with PP1α and PP1γ, but not PP1β (Fig. 1).

**Fig 2:**
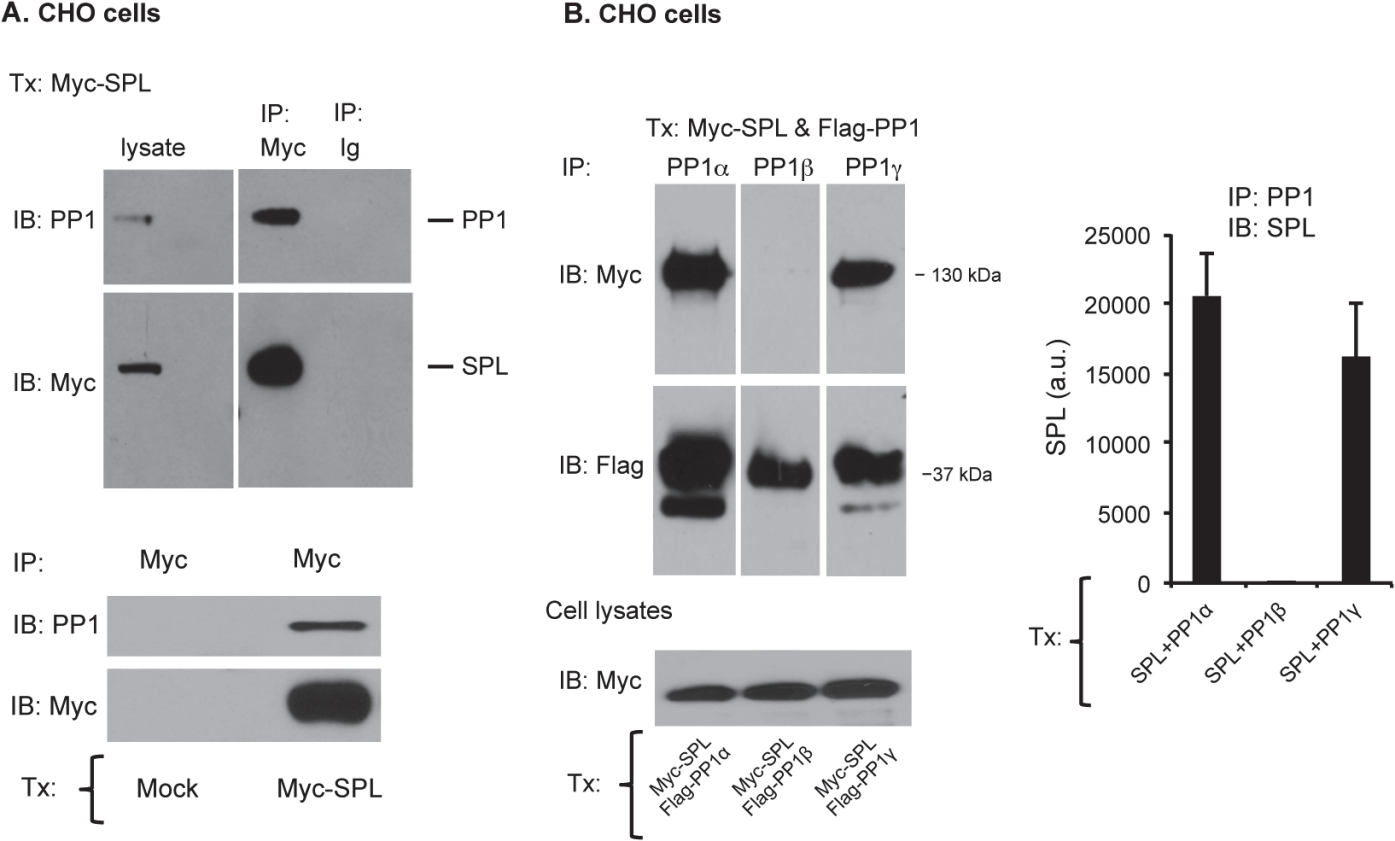
Formation of the SPL/PP1 complex in CHO cells. (A) *Top*: Lysates were prepared from CHO cells transfected with a full length, Myc-tagged spinophilin (Myc-SPL). Proteins were precipitated with an anti-Myc antibody or nonimmune immunoglobulin (Ig) and then probed for PP1 and Myc-SPL. *Bottom*: Lysates were prepared from mock-transfected control CHO cells or cells transfected with a full length, Myc-tagged spinophilin (Myc-SPL). Proteins were precipitated with anti-Myc and then probed for PP1 and Myc-SPL. (B) Lysates were prepared from CHO cells transfected with full length Myc-SPL and either PP1α, β or γ. Proteins were precipitated with PP1 isoform-specific antibodies and then probed for Myc-SPL or Flag-PP1 (mean ± SEM, N = 3). Note that all the samples were run on the same gel and, as indicated by the vertical gaps, two marker lanes were excised.

Both the binding of PP1 to spinophilin and the isoform selectivity for PP1 could be recapitulated in CHO cells. In the studies shown in Fig. 2, CHO cells were transfected with full length Myc-tagged spinophilin or mock-transfected as indicated. Immunoprecipitation with anti-Myc, but not with control IgG, precipitated PP1 as well as spinophilin from the Myc-spinophilin-transfected cells (Fig. 2A, *top*). Neither protein was precipitated with anti-Myc from mock-transfected cells (Fig. 2A, *bottom*). Reverse co-precipitation of spinophilin and PP1 was demonstrated in CHO cells co-transfected with Flag-tagged PP1α and Myc-SPL using anti-Flag to precipitate the proteins (S1 Fig.). Note, however, that a key difference between the CHO cell and platelet studies is that 1) CHO cells do not normally express SHP-1 [2] and 2) it was not necessary to stimulate the CHO cells with thrombin or SFLLRN in order to see an association of PP1α and PP1γ with spinophilin (Fig. 2B), an observation that proved useful in subsequent studies.

### Interactions between PP1, SHP-1 and spinophilin

**Fig 3:**
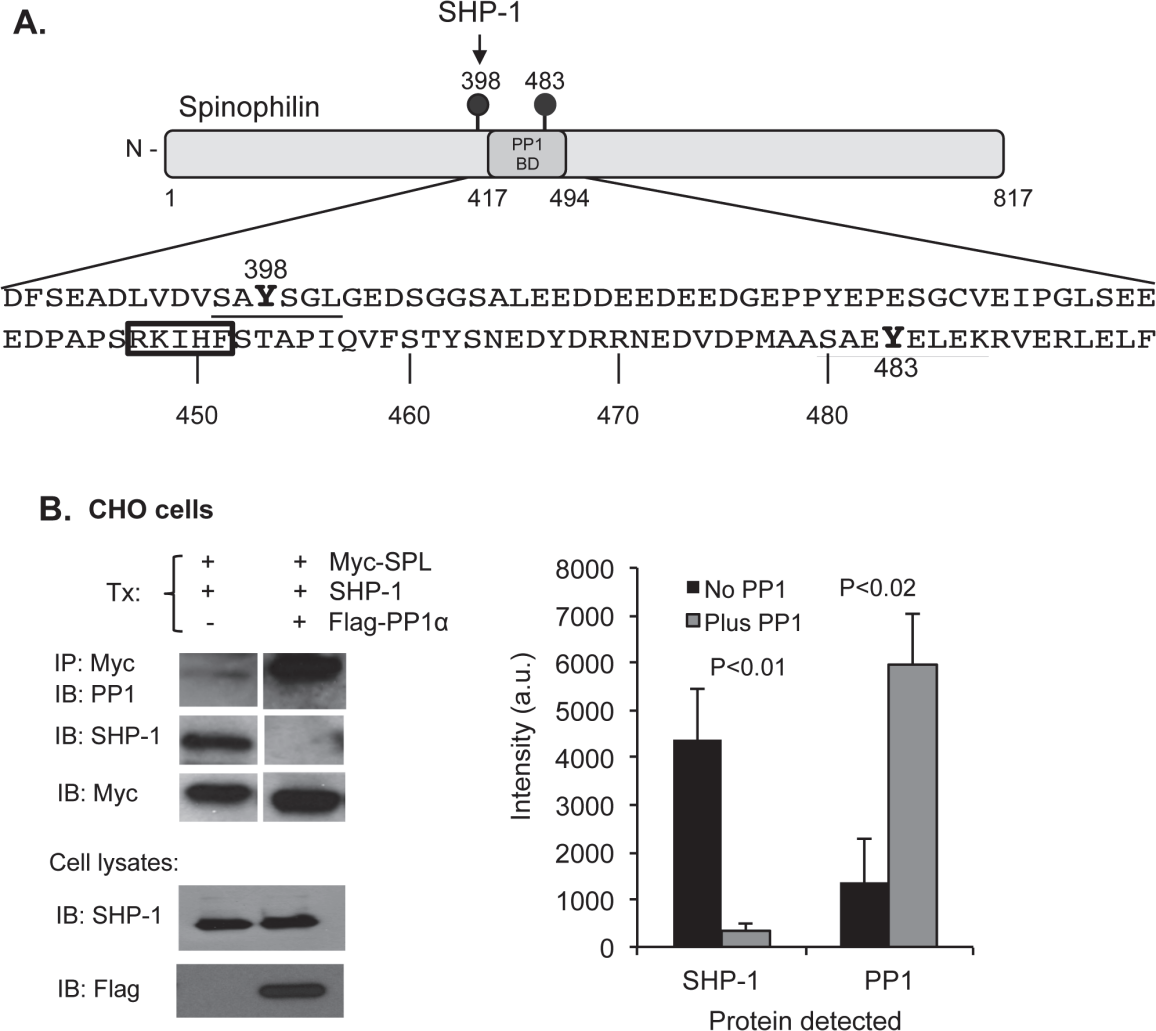
Competition of PP1 and SHP-1 for spinophilin. (A) A diagram of spinophilin showing the PP1 binding domain [31] and the SHP-1 binding site on phosphorylated Y398 [2]. There are two tyrosine phosphorylated residues in spinophilin: Y398 and Y483. The boxed RKIHF sequence matches a consensus sequence for PP1 binding (R/K)(R/K)(V/I)x(F/W) [27]. (B) *Top*: CHO cells were transfected with Myc-SPL, SHP-1 and Flag-PP1α as indicated. Proteins were precipitated with anti-Myc and probed for either SHP-1 or PP1. *Bottom*: Western blots of transfected cell lysates probed for SHP-1 or Flag-PP1α. The graph shows the amount of SHP-1 (left columns) or PP1 (right columns) that co-precipitated with SPL. For the black bars, only endogenous PP1 was present. For the gray bars, the cells were transfected with FLAG-PP1 (mean ± SEM, N = 5).

As illustrated in Fig. 3A, the binding site for PP1 on spinophilin has been mapped to residues 417 to 494 [31]. We have shown that a binding site for SHP-1 on spinophilin lies proximal to this region and includes SPL Y398 which, when phosphorylated, engages one of the two SH2 domains in SHP-1, an atypical arrangement that does not activate the phosphatase [2]. The proximity of the two binding regions suggested that in cells that express both PP1 and SHP-1, competition might occur between the two phosphatases for association with spinophilin.

Two sets of observations support this hypothesis. First, we examined the association of spinophilin with each of the two phosphatases in CHO cells, which normally express PP1, but not SHP-1. In CHO cells transfected with spinophilin and SHP-1 (but not PP1), SHP-1 binding to spinophilin was favored over endogenous PP1 (Fig. 3B, *left lane in the gel stack shown*). However, when PP1α was over-expressed, it displaced SHP-1 (Fig. 3B, *right lane*). Expression of Flag-PP1α had no effect on the expression of SHP-1 (Fig. 3B, bottom).

**Fig 4:**
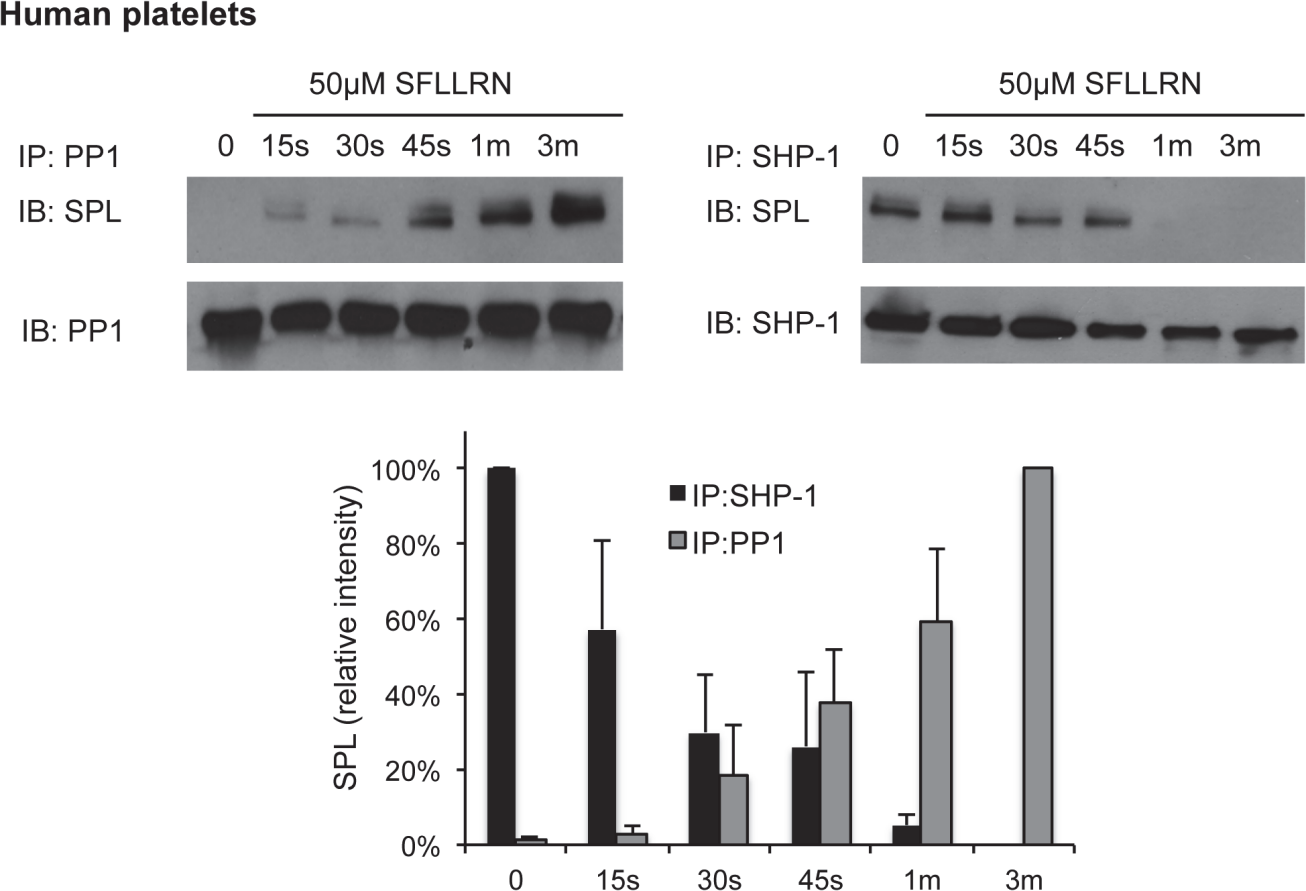
Temporal relationship between SHP-1 and PP1 binding to spinophilin in activated platelets. Human platelets were incubated with SFLLRN for each of the times indicated, after which proteins were precipitated with anti-pan-PP1 (E-9) or anti-SHP-1 and probed for spinophilin before being re-probed for PP1 or SHP-1 (mean ± SEM, N = 3).

Second, we compared the binding of endogenous SHP-1 and PP1 to spinophilin in platelets when the platelets were activated with SFLLRN. As already noted, we have previously shown that SHP-1 is associated with spinophilin in resting platelets, forming a complex with RGS10 and RGS18 that decays over approximately 1 minute when platelets are activated with SFLLRN [2]. Time course comparisons performed for the present studies show that the binding of PP1 to spinophilin increases as SHP-1 binding decreases, both events reaching completion 1 to 3 min after agonist addition (Fig. 4).

**Fig 5:**
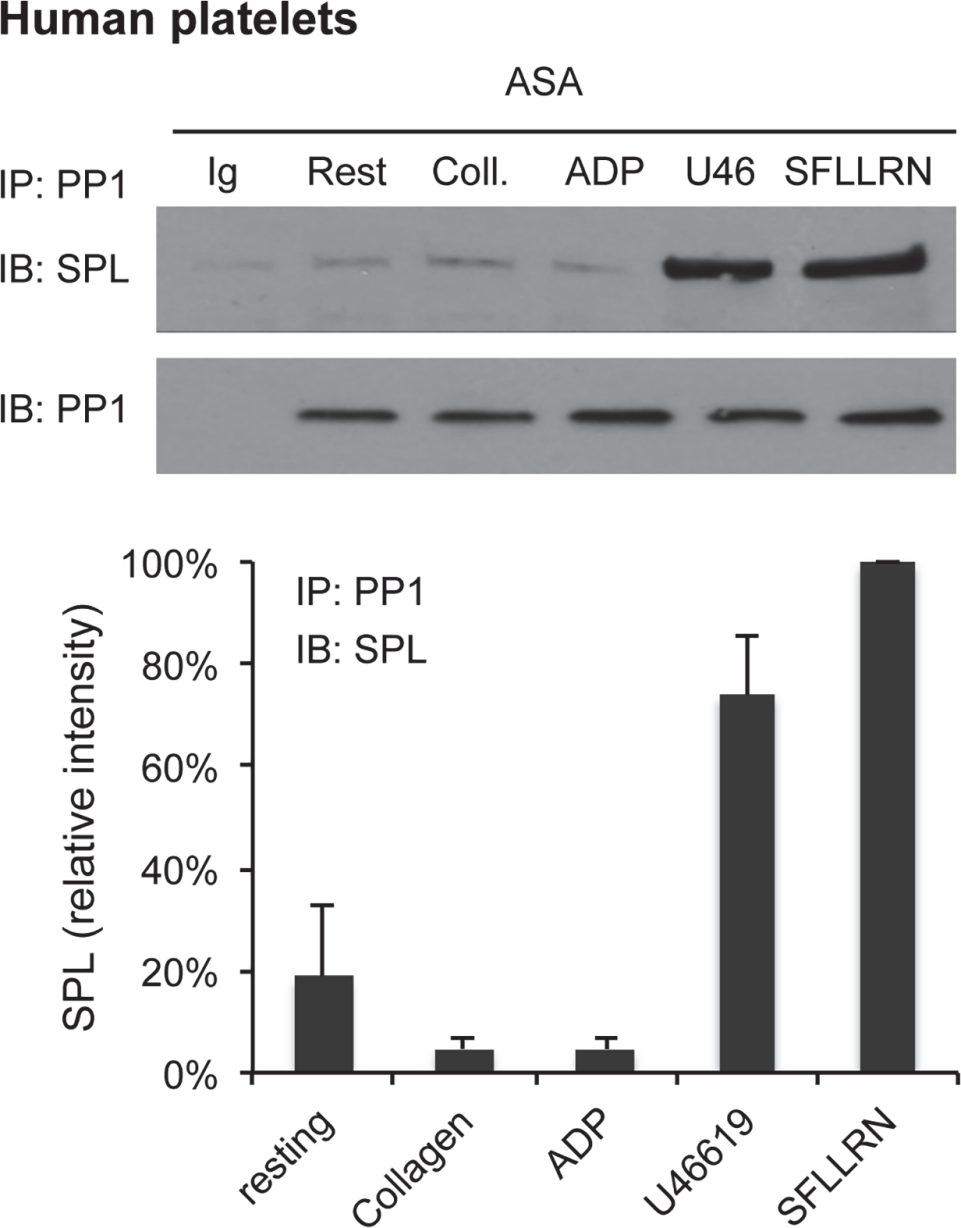
PP1 binding to spinophilin in platelets is agonist-selective. Human platelets were incubated for 3 min with 50 μM SFLLRN, a TxA_2_ mimetic (U46619 10μM), ADP (10μM) or collagen (10μg/ml) in the presence of 100 μΜ aspirin (ASA), after which the samples were lysed and placed on ice. Lysates were precipitated with anti-pan-PP1 (antibody E-9) and probed for spinophilin (antibody A-20) before being re-probed for PP1 (antibody FL-18). The results shown are the mean ± SEM, N = 4.

PP1 binding to spinophilin also increased when platelets were activated with the TxA_2_ mimetic, U46619. However, it did not increase when platelets were activated with either collagen or ADP when aspirin was added to inhibit endogenous TxA_2_ production (Fig. 5). This pattern of agonist selectivity is identical to the pattern we reported previously for dissociation of the SPL/RGS/SHP-1 complex and dephosphorylation of SPL Y398, both of which occur in response to thrombin and TxA_2_ mimetics, but not ADP or collagen [2]. These data show that 1) the binding of SHP-1 to spinophilin in favored in resting platelets and 2) suggest that the observed reciprocal differences in SHP-1 and PP1 binding during platelet activation reflect the departure of SHP-1 and the exposure of the PP1 binding site on spinophilin.

### Regulation of PP1 activity by spinophilin

Binding to spinophilin has been shown to inhibit PP1 activity although not with all substrates [27]. To understand the implications of the formation of the SPL/PP1 complex for an event that is relevant to platelet biology, we asked whether binding to spinophilin inhibits PP1 activity towards a small molecule substrate and then examined the effect of over-expressing spinophilin on myosin light chain (MLC) phosphorylation, comparing wild type spinophilin with a variant that is unable to bind to PP1. MLC is normally phosphorylated on Thr18 and Ser19 in platelets by a combination of myosin light chain kinase (MLCK) and Rho kinase, and dephosphorylated by PP1 [22]. Phosphorylation increases when platelets are activated, especially if a PP1 inhibitor is present [20, 32]. Our hypothesis was that if binding to spinophilin inhibits dephosphorylation of phospho-MLC by PP1, then the presence of wild type spinophilin should cause an increase in MLC phosphorylation, but a nonbinding variant should not.

**Fig 6:**
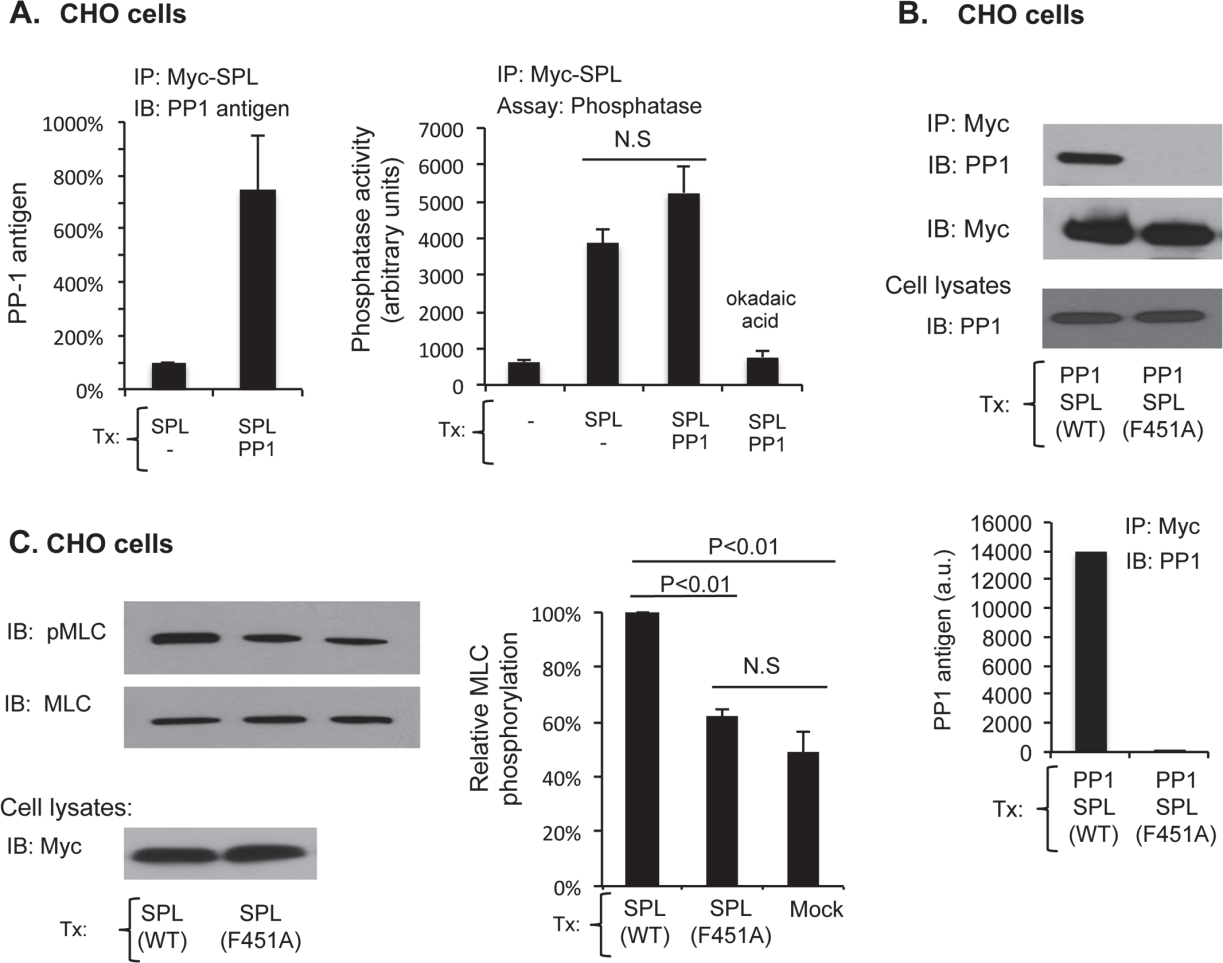
Binding to spinophilin inhibits PP1. (A) CHO cells were transfected with Myc-SPL and Flag-PP1α as indicated. Proteins were precipitated with anti-Myc and then probed with anti-PP1 (*left*) or assayed for SPL-associated phosphatase activity with a small molecule substrate (*right*) (mean ± SEM, N = 3). (B) CHO cells were transfected with PP1α and either Myc-tagged wild type or Myc-tagged F451A spinophilin as indicated. After immunoprecipitation with anti-Myc, precipitated proteins were probed with anti-PP1 and anti-Myc (mean ± SEM, N = 3). (C) *Top and right*. CHO cells were transfected with either wild type or F451A spinophilin and then immunoblotted for MLC and pMLC as indicated (mean ± SEM, N = 3). *Bottom*. Cell lysates were immunoblotted with anti-Myc, demonstrating equal expression of wild type and F451A spinophilin.

We began by co-expressing Myc-tagged SPL with Flag-tagged PP1α, immunoprecipitating spinophilin with anti-Myc and measuring PP1 antigen and phosphatase activity in the precipitate. In the studies summarized in Fig. 6A *left*, transfected CHO cell lysates were immunoprecipitated with anti-Myc and immunoblotted with anti-pan-PP1. There was an approximately 8-fold increase in PP1 antigen co-precipitating with spinophilin when the cells were co-transfected with PP1. In the studies shown in Fig. 6A *right*, precipitates formed with anti-Myc were assayed for phosphatase activity with a small molecule substrate. Expressing spinophilin was associated with an increase in phosphatase activity in the anti-Myc precipitates that was inhibitable with okadaic acid. There was, however, no further increase in phosphatase activity when the cells were co-transfected with PP1. Control studies showed that there was a large increase in phosphatase activity in anti-Flag precipitates from cells expressing Flag-PP1α, but no increase in activity in precipitates from cells expressing a catalytically-dead Flag-tagged PP1 variant (D95A) that acts as a dominant negative (S2 Fig.) [33]. Thus, these results show that co-expressing PP1 causes an increase in PP1 antigen associated with spinophilin, but not a corresponding increase in phosphatase activity measured with a small molecule substrate.

Over-expressing spinophilin also caused an increase in MLC phosphorylation detected with an antibody that recognizes pThr18 and pSer19. The comparator in this case was spinophilin in which the phenylalanine residue normally at position 451 had been replaced with alanine. F451 is located within the canonical PP1-binding sequence in spinophilin (see illustration in Fig. 3A). SPL F451A expresses as well as wild type spinophilin, but does not associate with PP1 (Fig. 6B). Over-expressing wild type spinophilin caused an increase in MLC phosphorylation relative to mock-transfected cells; over-expressing SPL F451A did not (Fig. 6C). Thus, it appears that binding to spinophilin can inhibit PP1 activity towards the small molecule substrate used in the phosphatase assays and towards phosphorylated myosin light chain in CHO cells.

## Discussion

Once initiated by vascular injury or vessel wall disease, platelet activation is regulated and vascular occlusion avoided by mechanisms that are incompletely understood. The goal in this study was to extend our earlier work on the SPL/RGS/SHP-1 complex by examining the timing and consequences of the interaction of PP1 with spinophilin. Our previous studies show that in resting platelets spinophilin is phosphorylated on tyrosines Y398 and Y483, forming a trimolecular complex with SHP-1 plus either RGS10 or RGS18. The role of pY483 is currently unknown, but pY398 provides an anchoring point for one of the two SH2 domains in SHP-1 [2].

**Fig 7:**
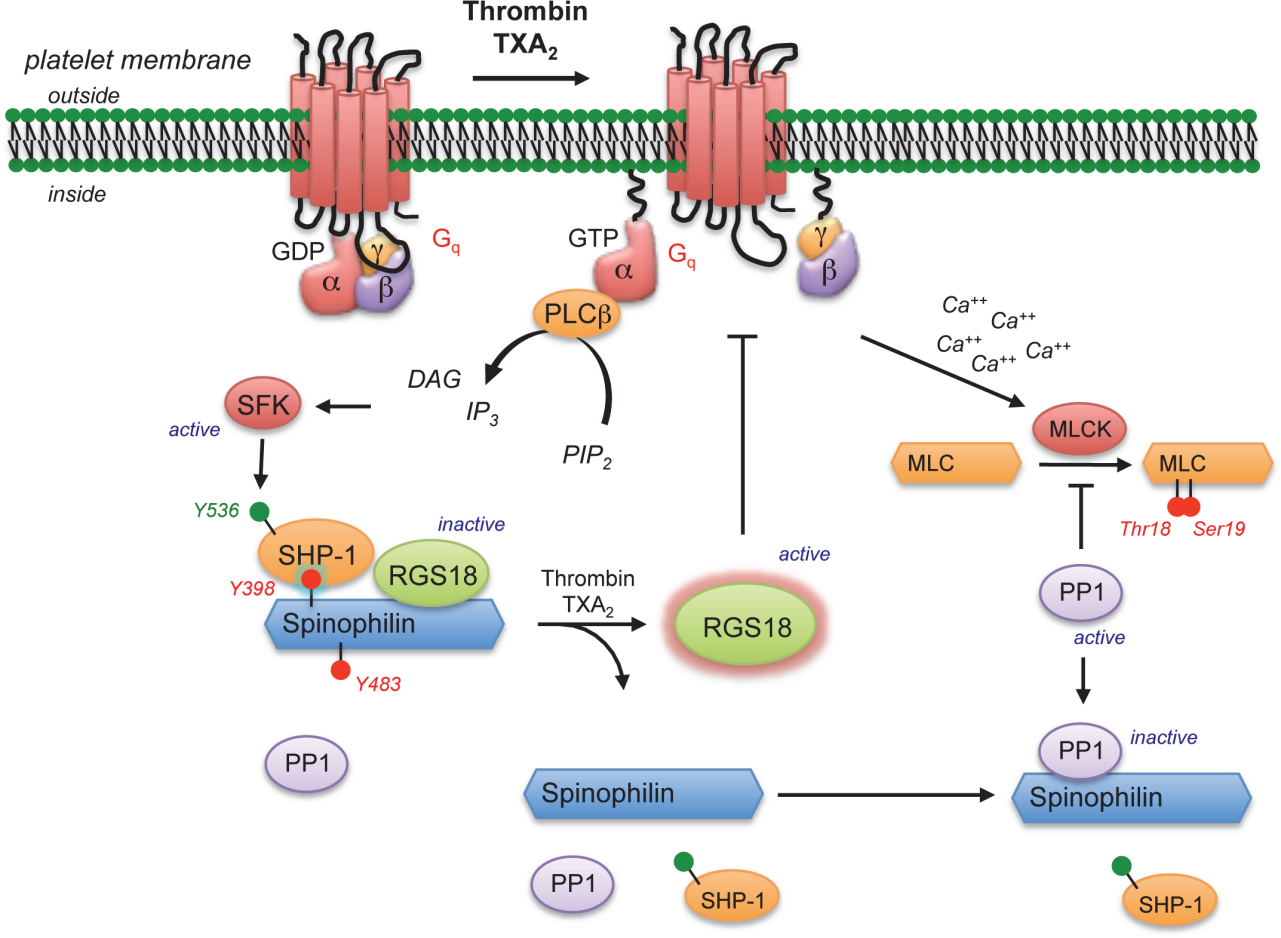
Agonist-induced dissociation of the SPL/RGS/SHP-1 complex and formation of the SPL/PP1 complex. In resting platelets spinophilin is phosphorylated on Y398 and Y483 and bound to either RGS10 or RGS18. Platelet activation by agonists for thrombin or TxA_2_ receptors activates SHP-1 by causing phosphorylation of SHP-1(Y536). This triggers dissociation of the SPL/RGS/SHP-1 complex, allowing RGS10 and RGS18 to inhibit signaling. Dissociation of SHP-1 from spinophilin is followed by binding of PP1. Binding to spinophilin inhibits the phosphatase, promoting phosphorylation of PP1 substrates such as myosin light chain (MLC).

When platelets are activated by thrombin and TxA_2_ mimetics, the SPL/RGS/SHP-1 complex becomes dephosphorylated and decays, gradually releasing SHP-1, RGS10 and RGS18. We have previously proposed that this delayed release of RGS proteins from spinophilin forms a negative feedback loop that limits the duration of subsequent G protein-dependent signaling (Fig. 7) [2]. Three lines of evidence support this conclusion. First, a mutation in Gi2α that renders it unable to bind to RGS10 and RGS18 results in a gain of function in platelets [7], as does the RGS18 knockout [9]. Second, knocking out spinophilin in mice impairs platelet function *in vitro* and *in vivo*, presumably due to a premature increase in RGS protein availability, and, third, introduction of a SHP-1 variant (Y536F) that can not be activated blocks thrombin-induced dissociation of RGS18 from spinophilin, producing a gain of function [2].

Thus, one consequence of the agonist-induced decay of the SPL/RGS/SHP-1 complex is that RGS proteins are released and become available to inhibit signaling by thrombin and TxA_2_ receptors. The second consequence of the decay of the SPL/RGS/SHP-1 complex is that spinophilin, now no longer phosphorylated on Y398 and Y483, becomes bound to PP1, inhibiting the phosphatase and promoting phosphorylation of PP1 substrates, including myosin light chain (Fig. 7). This positions spinophilin to play a role in regulating critical events such as shape change and clot retraction, both of which require contributions from platelet myosin [22–24]. These events do not occur simultaneously. On the time line of platelet activation and aggregation, clot retraction occurs relatively late. Getz, *et al*. have shown that the onset of myosin light chain phosphorylation accompanies shape change and that Gq-dependent phosphorylation of MLC Ser19 by MLCK persists longer than Thr18 phosphorylation [22]. Given the delay we observed between the onset of platelet activation and the formation of the SPL/PP1 complex, inhibition of PP1 by spinophilin may prove to be especially relevant for sustaining clot retraction, which in turn helps to stabilize the platelet/fibrin hemostatic mass.

PP1, in contrast to SHP-1, is broadly expressed and exists in 3 isoforms, all of which are expressed in platelets. Although its role in platelets is not fully understood, a large literature (mostly in cells other than platelets) indicates that PP1 has numerous regulatory/binding partners [34]. Given an abundance of options, the choice of binding partner(s) at any given time likely depends upon factors that include relative expression levels, relative binding affinities, intracellular localization and post-translational modifications. Our data indicate that in resting platelets, spinophilin preferentially binds to SHP-1 rather than PP1. The preference for SHP-1 presumably reflects in part the role that pY398 plays in anchoring SHP-1 to spinophilin [2]. Circumstances may prove different in cells other than platelets. SHP-1 expression is limited to hematopoietic cells and we have shown that the related and more widely-expressed nonreceptor tyrosine phosphatase, SHP-2, does not bind to spinophilin [2]. Although platelets express all 3 isoforms of PP1, we found that only PP1α and PP1γ were able to bind to spinophilin. The structural basis for this isoform selectivity is currently unknown.

How else might formation of the SPL/PP1 complex affect platelet activation beyond reducing the availability of PP1 to dephosphorylate myosin? There are multiple potential substrates and binding partners for PP1 in platelets. Quantitative proteomics data suggest that the number of copies of spinophilin is greatly exceeded by the number of copies of SHP-1, PP1, RGS10 and RGS18, both individually and collectively [4, 5]. If so, then it is likely that the location of spinophilin binding partners, as well as factors such as tyrosine phosphorylation and the presence of competitors plays an essential role in determining which copies of PP1 will be become bound to spinophilin and which PP1 substrates will be affected as a result. PP1 activity towards phosphorylated myosin light chains has been linked previously to a different PP1 binding protein, MYPT1 [35], whose copy number in platelets is similar to spinophilin [4]. The interrelations between PP1, MYPT1 and spinophilin remain to be explored, but it is of interest to note that PP1γ, which binds to MYPT1, is the one PP1 isoform that does not bind to spinophilin. This web of protein interactions within the broader platelet signaling network suggests a rich opportunity for regulating and tuning platelet activation events.

An interesting example of the larger relationships between signaling proteins, regulatory proteins and protein phosphatases is provided by recent publications on RGS18 phosphorylation. Gegenbauer and colleagues have shown that RGS18 in platelets is phosphorylated on Serine 49 and Serine 218, and that phosphorylation of Ser49 increases during platelet activation [10]. Phosphorylation on Ser49 and Ser218 allows RGS18 to bind to 14-3-3γ, one of a family of phosphoserine-binding scaffold proteins expressed in platelets. They have also shown that increased phosphorylation of RGS18 S216 in platelets exposed to PGI_2_ and forskolin (two inhibitors of platelet activation) induces dephosphorylation of S49 and S218 and provided evidence that this is mediated by PP1 [36]. Their data combined with our studies on the SPL/RGS/SHP-1 complex suggest an interesting handoff of RGS18 from spinophilin to 14-3-3γ during platelet activation, with consequences for RGS18 targeting and regulation [11]. We share their speculation that the binding of PP1 to spinophilin helps to preserve RGS18 S49 phosphorylation by inhibiting PP1, thereby stabilizing the RGS18/14-3-3 complex [36], and suggest that this effect might be expected to increase during platelet activation as more of the available pool of PP1α and PP1γ become bound to spinophilin. Given the reported differences in expression level of all of these molecules, sorting out their relationships in resting platelets, activated platelets, and platelets that have been rendered resistant to agonists by exposure to PGI_2_ or forskolin will prove to be a fruitful, if challenging, problem.

## Supporting Information

S1 Fig Precipitation controls and co-precipitation of spinophilin with PP1. CHO cells were transfected with Myc-SPL ± FLAG-PP1α as indicated. Protein were precipitated with either anti-FLAG or nonimmune immunoglobulin (Ig) and then probed for Myc-SPL and PP1 as indicated. Co-precipitation of SPL and PP1 is only seen when both are expressed. The experiment shown is representative of 2 similar studies.
(TIF)

S2 Fig Phosphatase assay. (A) CHO cells were transfected with either WT Flag-PP1α or Flag-PP1α (D95A), or were mock transfected. Afterwards the lysates were immunoprecipitated and phosphatase activity measured in the precipitate using a fluorogenic substrate (mean ± SEM, N = 3). (B) Immunoblot showing comparable expression of wild type and D95A spinophilin in transfected CHO cells.
(TIF)
