# Supplementary figures and images for: Dissociation of SHP-1 from Spinophilin during Platelet Activation Exposes an Inhibitory Binding Site for Protein Phosphatase-1 (PP1)
Source: PLoS One. 2015 Mar 18;10(3):e0119496. doi: 10.1371/journal.pone.0119496 (PMC4364895; doi:10.1371/journal.pone.0119496)

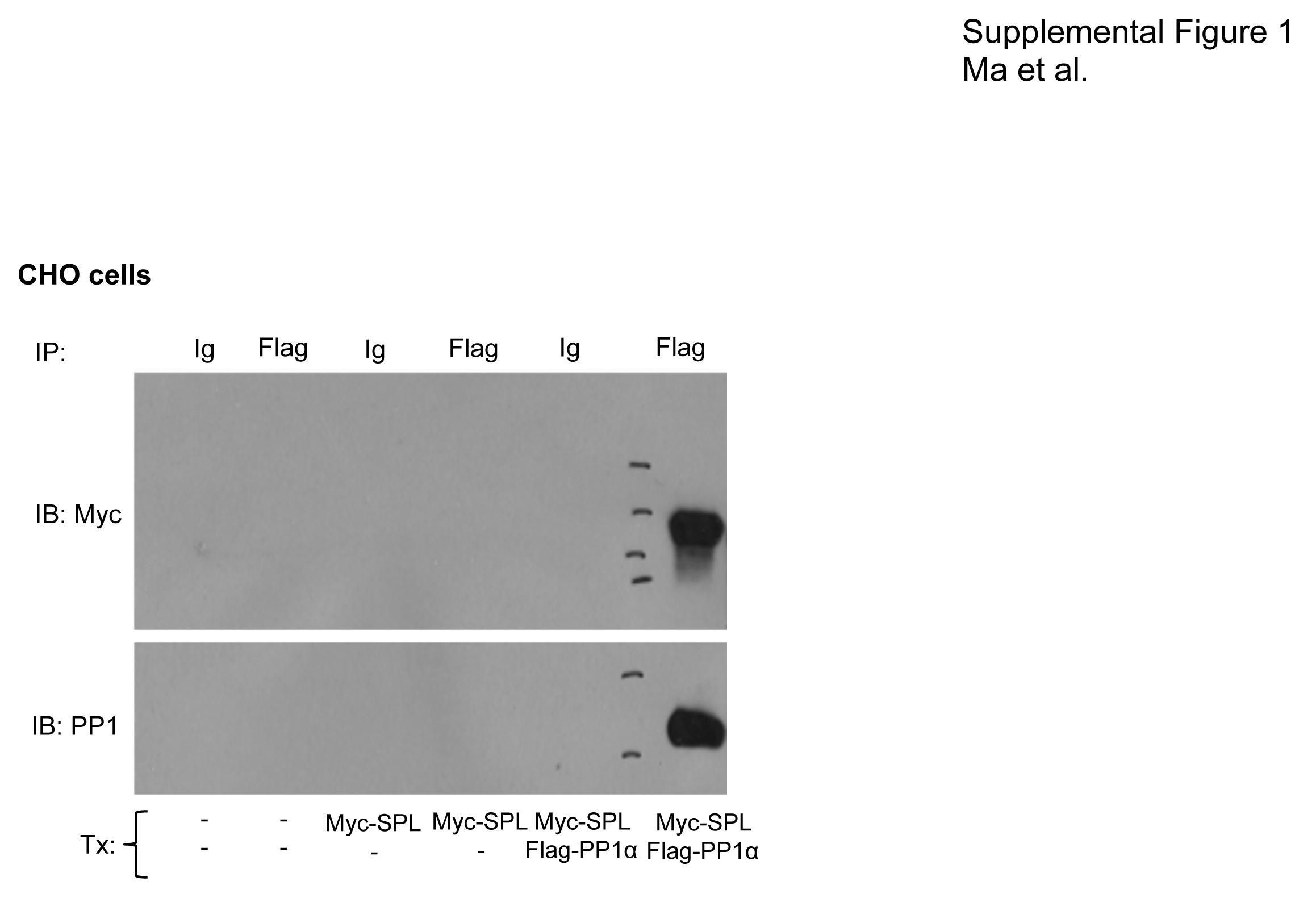

Supplement: S1 Fig — CHO cells were transfected with Myc-SPL ± FLAG-PP1α as indicated. Protein were precipitated with either anti-FLAG or nonimmune immunoglobulin (Ig) and then probed for Myc-SPL and PP1 as indicated. Co-precipitation of SPL and PP1 is only seen when both are expressed. The experiment shown is representative of 2 similar studies. (TIF) [file pone.0119496.s001.tif]

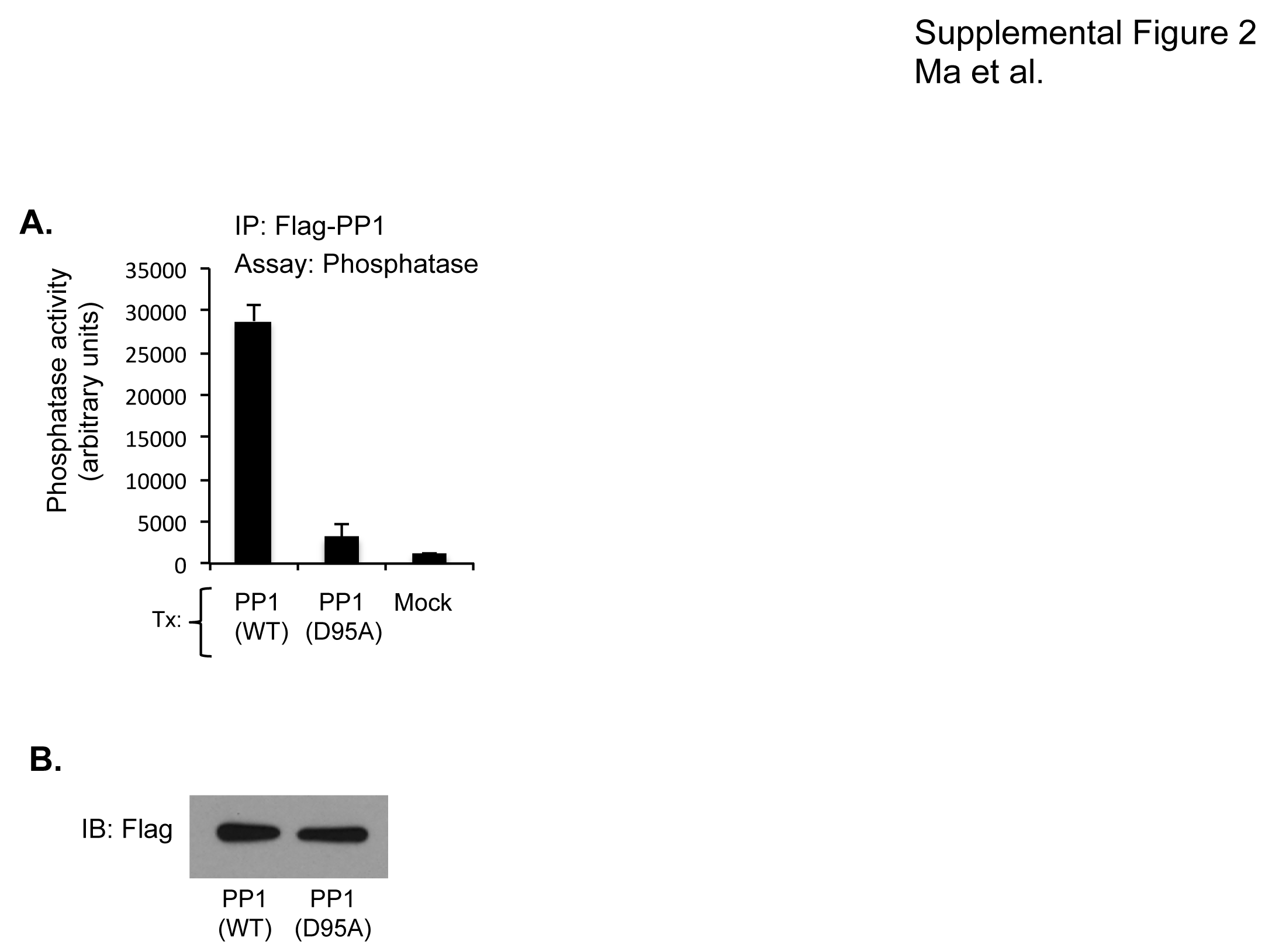

Supplement: S2 Fig — (A) CHO cells were transfected with either WT Flag-PP1α or Flag-PP1α (D95A), or were mock transfected. Afterwards the lysates were immunoprecipitated and phosphatase activity measured in the precipitate using a fluorogenic substrate (mean ± SEM, N = 3). (B) Immunoblot showing comparable expression of wild type and D95A spinophilin in transfected CHO cells. (TIF) [file pone.0119496.s002.tif]
